# Microcystin-LR Induces and Aggravates Colitis through NLRP3 Inflammasome-Mediated Pyroptosis in Mice

**DOI:** 10.3390/toxins15070447

**Published:** 2023-07-06

**Authors:** Yue Yang, Pan Gong, Xiuyan Long, Yuanjuan Jiang, Mingmei Ye, Sifan Tao, Yahui Su, Fei Yang, Li Tian

**Affiliations:** 1 Department of Gastroenterology, The Third Xiangya Hospital, Central South University, Changsha 410078, China; yangy930806@126.com (Y.Y.); zjjgp1234@163.com (P.G.); 13875990100@outlook.com (X.L.); 17320071569@163.com (M.Y.); 2204120719@csu.edu.cn (S.T.); 2 Hunan Province Key Laboratory of Typical Environmental Pollution and Health Hazards, School of Public Health, University of South China, Hengyang 421001, China; c06038440@126.com; 3 Xiangya School of Medicine, Central South University, 172 Tongzipo Road, Changsha 410078, China; 8301200121@csu.edu.cn; 4 Hengyang Medical School, The First Affiliated Hospital, University of South China, Hengyang 421001, China

**Keywords:** microcystin-LR, inflammatory bowel disease, NLRP3 inflammasome, pyroptosis

## Abstract

Inflammatory bowel disease (IBD) is a chronic, lifelong gastrointestinal disease, characterized by periods of activity and remission. The etiology of IBD is closely related to environmental factors. Previous studies have shown that the cyanotoxin microcystin-LR (MC-LR) causes intestinal damage, even IBD. To explore MC-LR’s effects and potential mechanisms on IBD occurrence and development, we used dextran-sulfate sodium gavage (DSS) and MC-LR together for the first time in mice. There were four groups of mice: (A) mice given PBS gavage (control, CT); (B) mice given 3% DSS gavage (DSS); (C) mice given 200 µg/kg MC-LR gavage (MC-LR); and (D) mice given 3% DSS + 200 µg/kg MC-LR gavage (DSS + MC-LR). Compared with the CT group, the MC-LR group and the DSS group demonstrated more severe colitis results, which presented as higher weight loss, an increased Disease Activity Index (DAI) score, shorter colon length, a higher degree of tissue structural damage, more apoptotic cells, and greater pro-inflammatory cytokines. Similarly, the DSS + MC-LR group showed more severe colitis compared with the DSS group. Subsequent experiments confirmed that MC-LR or DSS increased the expression of pyroptosis-related proteins mediated by the nucleotide-binding domain-like receptor protein 3 (NLRP3). Likewise, compared with the DSS group, the DSS + MC-LR group expressed these proteins at a higher level. In conclusion, our research is the first to show that MC-LR may induce colitis, and even IBD, through NLRP3 inflammasome-mediated pyroptosis, and it could aggravate DSS-induced colitis in the same way.

## 1. Introduction

Inflammatory bowel disease (IBD) is a chronic, recurrent, lifelong disease that mainly occurs in young adults aged 20 to 40, and it is known as a “green tumor”. Clinical manifestations typically include abdominal pain, diarrhea, and mucus bloody stools [1]. Research indicates that IBD patients’ colorectal cancer risk is time-dependent, increasing by 2% in ten years, 8% in twenty years, and 18% in thirty years [2]. It is estimated that by 2030, there will be nearly 4 million IBD patients in America [3]. Global epidemiological studies have indicated that the proportion of individuals with IBD is also gradually rising in newly industrialized regions, such as Africa, Asia, and South America [4]. IBD causes a lifetime burden [5] on individuals, caregivers, and society, including the direct costs of medicines, hospitalization, and surgery, and indirect costs, such as the loss of production capacity [6].

The specific etiology of IBD is still unclear, and current research views suggest that its pathogenesis is mainly related to individual genetic susceptibility, the external environment, intestinal microbiota, and individual immune response [7]. The epidemiological changes in IBD with the process of industrialization show that environmental factors are the risk factors of IBD [8,9]. Potential environmental factors that can contribute to IBD include food, drugs, hygiene, microorganisms, and pollution. Caused by water pollution and eutrophication, over 40% of European, Asian, and American lakes and reservoirs meet the favorable conditions for harmful algal blooms, of which 25–75% are considered toxic [10]. The growth of harmful algae can lead to the accumulation of microcystins (MCs). MCs are a type of heptapeptide toxin that not only has a negative impact on aquatic environments, but also constitutes an essential danger to human health [11]. One of the variants known for its high reproduction and severe toxicity is microcystin-leucine-arginine (MC-LR) [12]. Previous animal experiments have shown that the digestive system is a possible target for MC-LR toxicity, with some even suggesting that the gut is where MC-LR is most bioaccumulated [13]. Moreover, a study found that 1000 µg/kg MC-LR (the allowable level in drinking water is less than 1 µg/kg) could extend and exacerbate mouse colitis caused by 3% dextran sulphate sodium (DSS) [14], which has been widely used to model the disease features of IBD, and has been extensively validated in previous studies. Recently, research has indicated that continuous exposure to low levels of MC-LR causes disruption of the gut obstacle, chronic inflammation, fibrosis, and even IBD [15]. MC-LR is an effective inhibitor of serine/threonine-specific protein phosphatases (PPs), particularly PP1 and PP2A [16]. Inhibiting these enzymes alters normal protein activity, and causes cell harm, by upsetting the equilibrium between phosphorylation and dephosphorylation [17]. MC-LR has also been found to generate substantial reactive oxygen species (ROS) as part of its toxicological activity [18]. After the PPs’ suppression and oxidative stress, a succession of biochemical processes and signaling cascades starts, leading to intestinal damage. However, the mechanism by which MC-LR induces and aggravates IBD is unclear.

To clarify the impacts and the probable mechanisms of MC-LR on the onset and development of IBD, we conducted animal experiments co-treated by MC-LR and DSS for the first time, observed the inflammatory response, and then explored possible mechanisms. This work offers new perspectives into the effects of MC-LR in IBD, and presents new directions for the potential prevention and treatment of IBD.

## 2. Results

### 2.1. Body Weight and DAI Score

**Figure 1:**
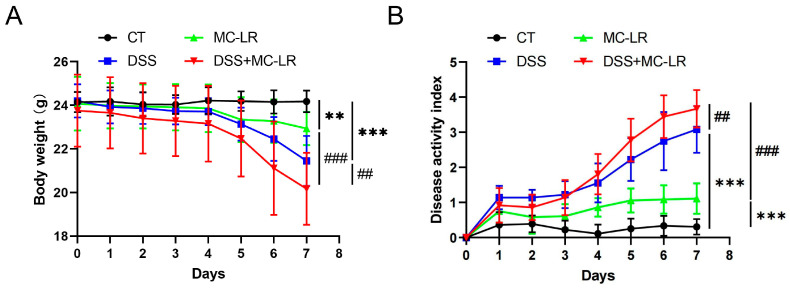
The effect of MC-LR and DSS on the body weight and DAI score. The body weight (**A**) and DAI score (**B**) were recorded every day. The data presented indicate the mean ± SD (one mice died in the DSS + MC-LR group, *n* = 11–12 mice per group). ** *p* < 0.01, *** *p* < 0.001 vs. CT group; ## *p* < 0.01, ### *p* < 0.001 vs. DSS + MC-LR group.

Throughout the exposure period, body weight, stools with blood, and the consistency of stool were all documented on a daily basis. One of the mice in the DSS + MC-LR group died during the experiment, and the rest survived. According to Figure 1A, the weight loss in the MC-LR (*p* = 0.008) and DSS (*p* < 0.000) groups was significantly greater than in the CT group. The DSS + MC-LR group lost more weight than the MC-LR (*p* < 0.000) and DSS (*p* = 0.008) groups. The Disease Activity Index (DAI) score of the mice indicated a decreased body mass, diarrhea, and severe bleeding. Similarly, MC-LR (*p* < 0.000) and DSS (*p* < 0.000) promoted the DAI score. Treatment with DSS + MC-LR could further promote DAI when compared with treatment with DSS (*p* = 0.007) or MC-LR (*p* < 0.000) alone (Figure 1B).

### 2.2. Colon Length

**Figure 2:**
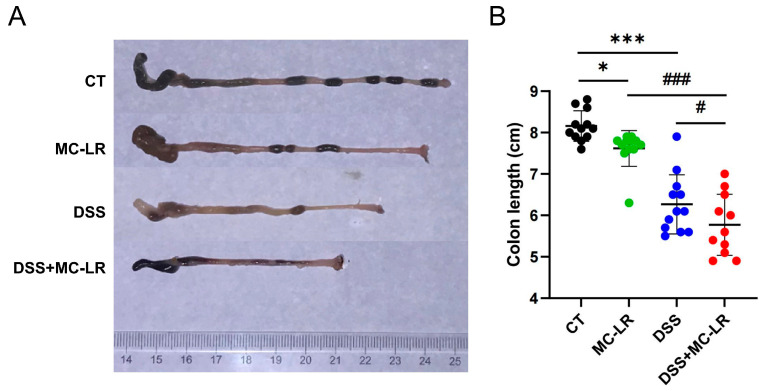
The effect of MC-LR and DSS on the colon length. (**A**) Representative macroscopic images of the mouse colons. (**B**) The quantitative lengths of colons. The data presented indicate the mean ± SD (one mouse died in the DSS + MC-LR group, *n* = 11–12 mice per group). * *p* < 0.05, *** *p* < 0.001 vs. CT group; # *p* < 0.05, ### *p* < 0.001 vs. DSS + MC-LR group.

The colon length represents the degree of colitis. The mice in the MC-LR (*p* = 0.028) and DSS (*p* < 0.000) groups had significantly shorter colons than the CT group. It was also discovered that the colons of the DSS + MC-LR group were shorter than those of the DSS (*p* = 0.049) and MC-LR (*p* < 0.000) groups (Figure 2).

### 2.3. Histological and Cytological Damage

**Figure 3:**
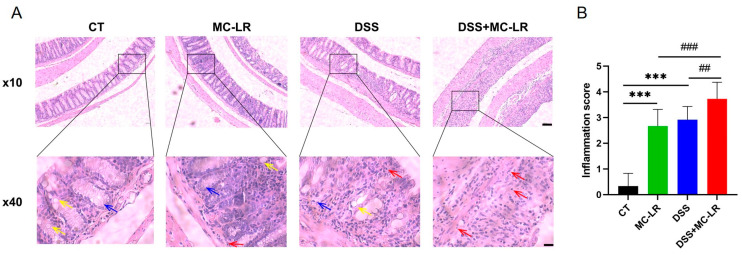
The effect of MC-LR and DSS on histopathological changes. (**A**) Representative H & E-stained images of colons. (**B**) The inflammation score. Red arrow, inflammatory cell infiltration; yellow arrow, goblet cell; blue arrow, crypt. Scale bar: 200 μm (up) and 50 μm (down). *** *p* < 0.001 vs. CT group; ## *p* < 0.01, ### *p* < 0.001 vs. DSS + MC-LR group.

**Figure 4:**
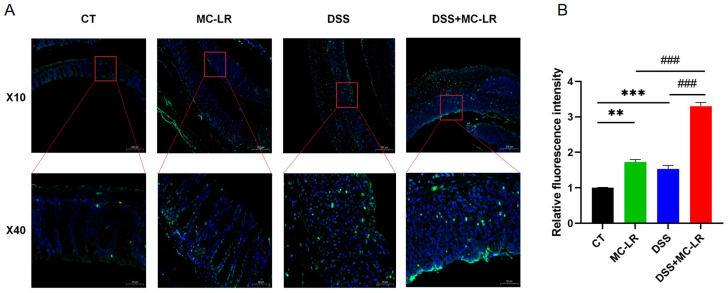
TUNEL staining was used to detect the mouse colonic tissue. (**A**) Fluorescence in green indicates a positive stain. (**B**) Relative fluorescence intensity. Scale bar: 200 μm (up) and 50 μm (down). ** *p* < 0.01, *** *p* < 0.001 vs. CT group; ### *p* < 0.001 vs. DSS + MC-LR group.

Clear structures, including intact mucosal epithelium, regularly arranged crypts, and numerous goblet cells, were observed in colons from the control (CT) group. Damaged structures, characterized by mucosal epithelial disruption, disorder of crypts, loss of goblet cells, and inflammatory cell infiltration, were observed in colons from the MC-LR group and DSS group. Seriously damaged structures, including mucosal epithelial collapse, almost disappeared crypts, and goblet cells, were observed in colons from the DSS + MC-LR group (Figure 3). Moreover, we detected a number of broken DNA fragments of cells in the colonic tissues via TUNEL staining. Compared to the CT group, the number of injured cells in the colon was significantly higher in the DSS and MC-LR treatment groups. Furthermore, compared to the DSS group, the number of injured cells was significantly higher in the DSS + MC-LR group. (Figure 4).

### 2.4. The Expression Levels of Pro-Inflammatory Cytokines

**Figure 5:**
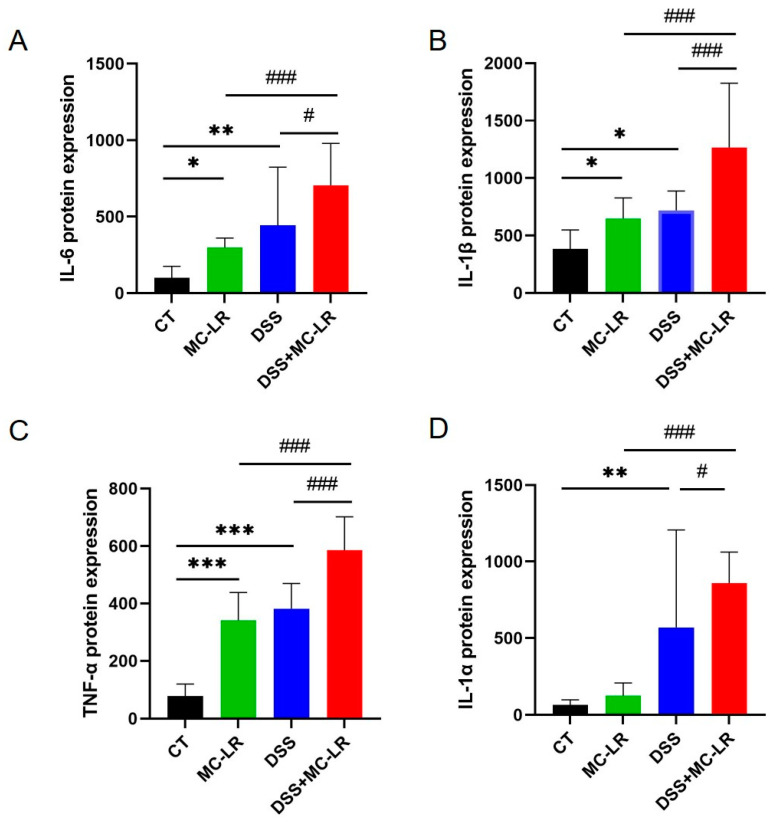
The effect of MC-LR and DSS on pro-inflammatory cytokines. The protein level of the IL-6 (**A**), IL-1β (**B**), TNF-α (**C**), and IL-1α (**D**) in colonic tissue was detected using ELISA. The data are expressed as mean ± SD (one mouse died in the DSS + MC-LR group, *n* = 11–12 mice per group). * *p* < 0.05, ** *p* < 0.01, *** *p* < 0.001 vs. CT group; # *p* < 0.05, ### *p* < 0.001 vs. DSS + MC-LR group.

The expression of several cytokines in the colonic tissue isolated from the mice was detected through ELISA, to evaluate the impact of MC-LR and DSS-induced colitis. In the DSS and MC-LR treatment groups, the levels of IL-6 (DSS: *p* = 0.001; MC-LR: *p* = 0.048), IL-1β (DSS: *p* = 0.011; MC-LR: *p* = 0.043), and TNF-α (DSS: *p* < 0.000; MC-LR: *p* < 0.000) were increased, relative to the CT group. Furthermore, when compared to the DSS and MC-LR groups, the DSS + MC-LR group had higher levels of IL-6 (DSS: *p* = 0.012; MC-LR: *p* < 0.000), IL-1β (DSS: *p* < 0.000; MC-LR: *p* < 0.000), TNF-α (DSS: *p* < 0.000; MC-LR: *p* < 0.000), and IL-1α (DSS: *p* < 0.000; MC-LR: *p* < 0.000) (Figure 5). MC-LR increased the expression of IL-6, IL-1β, TNF-α, and IL-1αin in the DSS-induced colitis model, thereby aggravating the inflammation.

### 2.5. NLRP3-Related Proteins

**Figure 6:**
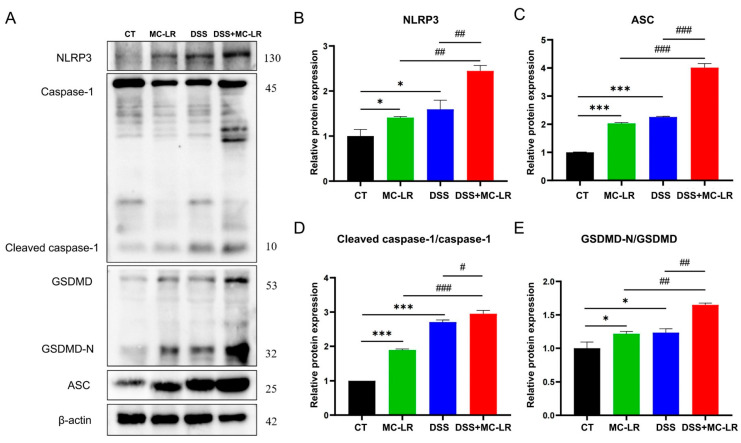
The effect of MC-LR and DSS on NLRP3-mediated pyroptosis-related proteins. The immunoblot analysis (**A**) of NLRP3 and other indicated proteins, including ASC, caspase-1, GSDMD, GSDMD-N, cleaved caspase-1, and the relative expression to the control group (**B**–**E**). The data are expressed as mean ± SD (one mouse died in the DSS + MC-LR group, *n* = 11–12 mice per group). * *p* < 0.05, *** *p* < 0.001 vs. CT group; # *p* < 0.05, ## *p* < 0.01, ### *p* < 0.001 vs. DSS + MC-LR group.

The expression of IL-1β was elevated in the DSS + MC-LR group, and the nucleotide-binding domain-like receptor protein 3 (NLRP3) inflammasome was the primary origin of IL-1β in the mucosa [19]. To clarify more potential pathways of MC-LR in the initiation and advancement of IBD, we explored the expression levels of NLRP3-mediated pyroptosis-related proteins. Western blot (WB) was used to determine the protein expression levels of NLRP3, apoptosis-associated speck-like protein containing a caspase recruitment domain (ASC), caspase-1 cleavage, and gasdermin D (GSDMD). When compared to the CT group, the expression of NLRP3 (DSS: *p* = 0.013; MC-LR: *p* = 0.043), ASC (DSS: *p* < 0.000; MC-LR: *p* < 0.000), caspase-1 cleavage (DSS: *p* < 0.000; MC-LR: *p* < 0.000), and GSDMD (DSS: *p* = 0.017; MC-LR: *p* = 0.022) was significantly greater in the MC-LR and DSS treatment groups. Again, the expression of NLRP3 (DSS: *p* = 0.004; MC-LR: *p* = 0.002), ASC (DSS: *p* < 0.000; MC-LR: *p* < 0.000), caspase-1 cleavage (DSS: *p* < 0.016; MC-LR: *p* < 0.000), and GSDMD (DSS: *p* = 0.002; MC-LR: *p* = 0.002) in the DSS + MC-LR group showed significantly higher levels than in the MC-LR or DSS groups. (Figure 6).

## 3. Discussion

As a lifelong disorder characterized by relapses, IBD can impose a substantial burden on society [5]. Although its specific etiology is not clear, epidemiological investigations have shown that the onset of IBD has a strong connection to environmental factors [8]. MC-LR is an environmental toxin widely present in water bodies and food; the World Health Organization (WHO) has established a maximum MC-LR concentration in drinking water of 1 g/L [20]. However, studies have found that the peak concentration of MC-LR in artificial ponds in the Yangtze River Delta region of China could reach 40.6 μg/L, and in Southern Africa, the concentration of MC-LR in Mozambican drinking water ranges from 6.83 to 7.78 μg/L. MC-LR is becoming more common and severe in freshwater systems around the world [21]. The concentration of MC-LR in numerous water sources far exceeds the WHO recommendation [22]. It enters our bodies mostly through the consumption of contaminated food and water, causing multi-organ damage [12]. Previous studies have found that persistent contact with low concentrations (1, 60 and 120 μg/L) of MC-LR leads to intestinal barrier breakdown, chronic inflammation, fibrosis, and even IBD [15]. Nevertheless, the mechanism by which MC-LR induces IBD and aggravates colitis is unknown. At present, most studies on the relationship between environmental factors and the development of IBD have explored further environmental factors on the basis of the DSS-induced colitis model. However, the influence of environmental factors usually occurs over the whole process of disease occurrence, rather than only after the occurrence of IBD. Therefore, we have improved the existing treatment method, and constructed an animal model that is more in line with the occurrence and development process of IBD, by treating environmental factors and DSS at the same time, so as to explore the role and mechanism of environmental factors in the occurrence and development of IBD.

Compared with the CT group, a colitis model was successfully constructed in the DSS group, as evidenced by higher weight loss, an increased DAI, shorter colon length, more serious colonic tissue damage, and higher levels of pro-inflammatory cytokines (IL-6, IL-1β, TNF-α, and IL-1α). It was consistent with earlier research [23]. In comparison to the CT group, the MC-LR group’s results showed higher weight loss, an increased disease DAI, shorter colon length, more serious colonic tissue damage, and increased pro-inflammatory cytokine levels (IL-6, IL-1β, and TNF-α). Our previous research demonstrated that chronic low-level MC-LR (1, 60 and 120 μg/L through water consumption) could induce intestinal damage, and even IBD [15]. In our research, animals were exposed to higher doses of MC-LR (gavaged with 200 µg/kg) for a short phase. The two experimental designs showed similar results. Moreover, similar results were obtained for the MC-LR group and the DSS group. All of these indicate that MC-LR may induce colitis, and even IBD. The MC-LR + DSS group was more severely affected by colitis in comparison to the DSS group, as evidenced by greater weight loss, an elevated DAI score, a decreased gut length, a higher degree of tissue structural damage, more apoptotic cells, and higher levels of IL-6, IL-1α, IL-1β and TNF-α in the colonic tissue. This suggests that MC-LR aggravates DSS-induced colitis. As reported by Su et al., MC-LR exacerbates DSS-induced colitis, and prolongs its duration [24]; in their research, MC-LR was used in an animal model of pre-existing IBD. However, environmental factors (i.e., MC-LR) have been at work over the entire course of IBD, including the stages of induction and progression. Furthermore, the concentration of MC-LR is also a concern for us. The estimated daily intake (EDI) of the World Health Organization limit is 0.04 µg MC-LR/kg/day. Although the actual concentration of MC-LR in water cannot reach this level, MC-LR can enter the human body through a variety of routes, including contaminated drinking water; aquatic, terrestrial animal, and cyanobacteria dietary supplements; contaminated vegetables and fruits irrigated with LR-containing water; inhalation and skin exposure during water recreation; and intravenous hemodialysis with contaminated water. Via the aforementioned routes, MC-LR accumulates in the human body. Existing studies have shown that the adult EDI of MCs in plants ranges from 3480–3630 ng/kg/d, both of which are 87–90-fold higher than the WHO detection limit [25], and the mean concentrations of MCs in the kidney, liver, and muscle of the silver carp were 45.1, 40.0, and 29.1 ng/g, dm, respectively. However, in the presence of dense cyanobacteria blooms, the mean concentrations of MCs in the kidney, liver, and muscle of the silver carp were 782, 957, and 197 ng/g, dm, respectively. The concentrations of MC-LR were chosen for this study according to WHO guidelines and peer literature. Su et al. gave mice 1000 µg/kg of MC-LR to investigate the impact of MC-LR on the severity of colitis [14,24]. By administering 200 µg/kg of MC-LR intragastrically to mice, Chen et al., discovered that MC-LR treatment could drastically alter the structure of the mice’s intestinal bacteria, causing intestinal injury [26]. Our previous research (unpublished) found inflammation and fibrosis in colon tissue in mice, resulting from a 21-day intraperitoneal injection of 20 µg/kg MC-LR. The intraperitoneal injection of 20 µg/kg MC-LR was approximately similar to the treatment dose of 200 µg/kg MC-LR administered by gavage, according to the conversion of bioavailability [27]. Therefore, in this study, we selected a dose of 200 μg/kg MC-LR for the gavage treatment of mice, as we thought that for the onset process of IBD, this experimental concentration selection was appropriate [28]. In short, we used novel approaches for animal modeling: mice were simultaneously given MC-LR and DSS, instead of DSS treatment before MC-LR exposure [14]. We believe this modeling method is more consistent with MC-LR’s mode of action in IBD. Therefore, the experimental results better reflect the role of MC-LR in IBD.

IL-6, IL-1α, IL-1β, and TNF-α are common pro-inflammatory cytokines that cause inflammation in IBD patients [29]. Among these factors, TNF-a and IL-1β play an essential function in the development of IBD. TNF-a is a key cytokine that damages the intestine by activating intracellular signaling pathways, such as NF-kB, MAPK, and caspases [30]. In addition, anti-TNF-a agents have become effective medications for IBD [31]. In the results of this research, the expression of TNF-a was significantly increased. This indicated that MC-LR might facilitate the occurrence and development of IBD. IL-1β comprises alarmins in inflammasomes, eliciting systemic inflammation [32]. Previous research has shown that a raised IL-1βexpression is linked with an elevated IBD severeness [33]. Furthermore, the mucosal IL-1β was primarily produced by the NLRP3 inflammasome [19]. We discovered that the IL-1β levels were significantly elevated, thus we examined the levels of NLRP3-mediated pyroptosis-related proteins.

**Figure 7:**
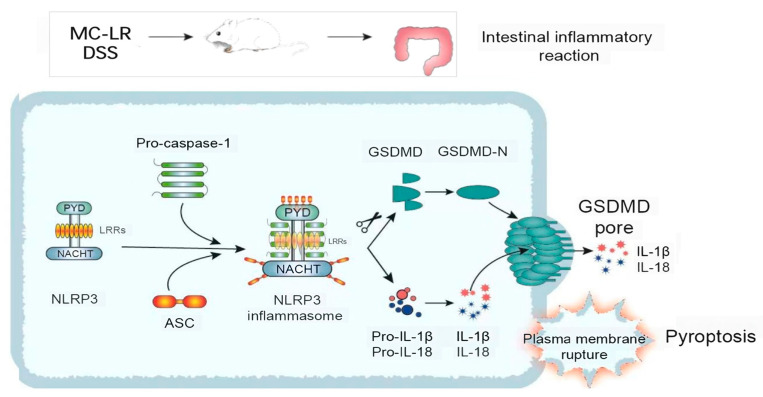
The mechanism by which MC-LR promotes the occurrence and development of IBD.

The NLRP3 inflammasome is crucial to the pathogenesis and progression of IBD [34]. Current study evidence has shown that large amounts of the NLRP3 inflammasome exacerbate colitis [35]. In addition, the lack of NLRP3 inflammasome protects against DSS-induced colitis [36]. Our previous research proved that inhibiting the NLRP3 pathway can reduce inflammation in IBD animal models [37,38]. The NLRP3 inflammasome is a protein complex made up of NLRP3, apoptosis-associated speck-like protein with CARD (ASC), and caspase-1 [39]. A diverse range of microbial substrates, signaling molecules, and environmental stimuli may activate the NLRP3 receptor. The action process of the NLRP3 inflammasome is divided into two stages: priming and activation. Firstly, the expression of NLRP3, pro-IL-1β, and pro-IL-18 is increased in response to various irritants, just like toll-like receptor ligands. Secondly, during the activation step, NLRP3 inflammasome assembly starts to respond to damage signals, such as transcellular ion flux, damaged lysosomes, and damaged mitochondria, causing the cleavage of pro-caspase-1. The active caspase-1 (cleaved caspase-1) induces the activation of IL-1β and IL-18, as well as the pore-forming protein GSDMD-N, by cleaving their precursors (Figure 7). In our work, the results demonstrate that the level of NLRP3, ASC, the cleavage of caspase-1, and GSDMD were considerably elevated in the MC-LR and DSS groups, compared to the CT group. Similarly, the level of these proteins was markedly increased in the DSS + MC-LR group, compared to the MC-LR or DSS group. The MC-LR facilitated the level of NLRP3 in the colonic tissue of colitis mice, suggesting that the digestive cytotoxicity of MC-LR in IBD is related to the priming of the NLRP3 inflammasome. IL-1β, cleaved caspase-1, and GSDMD-N were detected in the colonic tissue, and these findings suggest that MC-LR intestinal toxicity in IBD is associated with NLRP3 inflammasome assembly. In addition, GSDMD-N is the executor of pyroptosis [40], implying that MC-LR may promote pyroptosis in the colons of IBD patients.

## 4. Conclusions

Through our new, improved animal model, we explored the effects and the preliminary mechanisms of MC-LR on the occurrence and development of IBD. The results first revealed that MC-LR, through NLRP3 inflammasome-mediated pyroptosis, may induce colitis and even IBD, and it could aggravate DSS-induced colitis in the same way. This could deepen the understanding of the specific mechanism by which MC-LR facilitates the occurrence and development of IBD, and provide potential new directions for the prevention and therapeutic target of IBD in the future.

## 5. Materials and Methods

### 5.1. Reagents and Antibodies

DSS was purchased from MP Biomedicals (Santa Ana, CA, USA). MC-LR with a purity of 95% was purchased from Alexis Corporation (Lausen, Switzerland). The IL-1β ELISA kits, IL-6 ELISA kits, TNF-α ELISA kits and IL-1α ELISA kits were purchased from Invitrogen Life Technologies (Carlsbad, CA, USA). The Tunel kits was purchased from Beyotime (Shanghai, China). The IL-1β antibody was purchased from RD System (Minneapolis, MN, USA). The caspase-1 antibody and GSDMD antibody were purchased from Abcam (Cambridge, UK). The ASC antibody was purchased from Adipogen (San Diego, CA, USA). The β-actin antibody was purchased from Cell Signaling Technology (Danvers, MA, USA).

### 5.2. Mice

Hunan SJA Laboratory Animal Co., Ltd. (Hunan, China) provided SPF male C57BL/6 mice that were between 20 and 22 g, and eight weeks old. The mice were raised in China at the Experimental Animal Centre of Central South University. They were fed standard mouse pellets, and kept in a room with 12 h of light and 12 h of darkness. All experiment plans were accepted by the Animal Care and Use Committee at Central South University (permit no. XYGW-2018–41). Serum and colon tissues were gathered for the studies 24 h following the last day.

### 5.3. Modeling and Experimental Design

**Figure 8:**
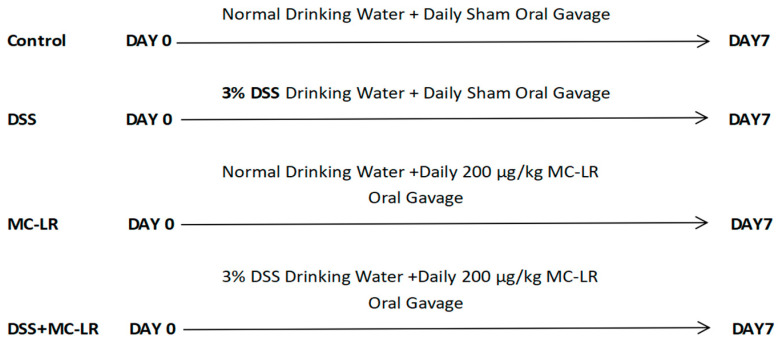
Experimental Design Diagram.

After seven days of acclimatization and feeding, 48 mice were randomized into four groups (*n* = 12 in every group): (A) mice given PBS gavage (control, CT); (B) mice given Dextran-sulfate sodium gavage (DSS); (C) mice given MC-LR gavage (MC-LR); and (D) mice given DSS + MC-LR gavage (DSS + MC-LR). In group A, mice were permitted to consume water ad libitum for 7 days, while also receiving PBS (0.2 ml) via sham oral gavage; in group B, mice were given the freedom to consume water containing 3% DSS for 7 days, and were also orally gavaged with PBS daily; in group C, mice were permitted to consume water ad libitum for 7 days, and were also gavaged with 200 µg/kg MC-LR daily; in group D, mice were given the freedom to consume water containing 3% DSS for 7 days, and were also gavaged with 200 µg/kg MC-LR daily (Figure 8). Body weight, feces, and body posture were measured every day during the trial, to calculate the DAI [36,41]. The DAI is the average number of the weight loss compared to the starting weight, the consistency of the stool, and blood. At the designated time points, mice were killed, and the sample was removed instantly for length evaluation, tissue culture, Western blotting analysis, and histological assessment.

### 5.4. Histology

All mice were euthanized on day 7, and the colon was measured next to a standard ruler, and photographed. Immediately, the collection and analysis of the colorectal samples was performed. The bloodstains were rinsed away with PBS (pH = 7.2). Next, a segment of distal colon (1.0 cm) was rolled, and then preserved overnight with 4% paraformaldehyde (PFA) in sterile PBS buffer at room temperature. Paraffin was used to implant the tissues. HE was used to stain 4 m-thick sections of tissue that had been dewaxed. An optical microscope (Motic, BA210) was then employed, to inspect and photograph the slices.

### 5.5. Enzyme-Linked Immunosorbent Assay (ELISA)

The ELISA double-antibody sandwich method was used to measure inflammatory cytokines (IL-1β, IL-6, TNF-α, and IL-1α). The test technique and statistics strictly adhered to the kit’s instructions. Several 450 nm detectors (BioTek, Winooski, VT, USA) were used in a microplate reader, to determine the absorbance value. The inflammatory cytokine concentration is proportional to the OD_450_ value, and the inflammatory cytokine concentration in the sample can be calculated by drawing a standard curve.

### 5.6. Western Blotting (WB)

From colorectal tissues that had been lysed in ice-cold RIPA solution, total protein was extracted (Beyotime, Shanghai, China). The BCA technique was utilized to measure the protein concentration (Bio-Rad, Hercules, CA, USA). SDS-PAGE was used to separate the proteins, then the separated proteins were electroblotted onto a polyvinylidene fluoride (PVDF) membrane (Merck Millipore Ltd., Burlington, MA, USA). Protein Free Rapid Blocking Buffer (EpiZyme Biotechnology, Shanghai, China) was used to block the transmitted membrane. Antibodies were used to treat the membranes overnight at 4 °C. These antibodies included anti-NLRP3, anti-GSDMD, anti-caspase-1, anti-ASC, and anti-β-actin. The transplanted membrane was incubated for 60 min with goat anti-mouse IgG (H + L) HRP conjugate or goat anti-rabbit IgG (H + L) HRP conjugate. Protein bands were detected and quantified using the Bio-Rad chemiluminescence imaging system, and Millipore’s Luminata Forte Western HRP substrate. The intensity of the band was measured using ImageJ (Rawak Software, Inc. Munchen, Germany).

### 5.7. TUNEL Staining

The slices were dewaxed with xylene and anhydrous ethanol successively, then washed with distilled water. After the slices were slightly dried, the circle was dripped with protease K (Beyotime, ST538) working solution, and incubated at 37 °C for 22 min. Then the slices were placed in PBS (AIFang blological, AFIHC018) (PH7.4) and shaken three times for five minutes using the decolorizing shaker. When the slices were slightly dried, the working solution for breaking the membrane (Servicebio, G1204) was added into the circle, and incubated for 20 min at room temperature. The slices were then washed three times for five minutes each in PBS, using the decolorizing shaker. The appropriate amount of TDT enzyme was taken from the TUNEL kit, mixed with dUTP at a ratio of 1:49, and added to the covered tissue in the circle. Slices were placed flat in the wet box, incubated at 37 °C for 2 h, and a small quantity of water was added to the wet box to maintain humidity. The slices were washed with PBS three times, each time for 5 min. After the PBS was removed, the circle was stained with DAPI (Beyotinme, C1002) for 10 min at room temperature. The slices were then washed three times for five minutes each in PBS, using the decolorizing shaker. The slices were slightly dried and sealed with anti-fluorescence quenching tablets. Images were taken while the slices were being observed under a fluorescence microscope. DAPI is excited by ultraviolet light with a wavelength of 330–380 nm, and it gives off a blue light. FITC is excited by ultraviolet light with a wavelength of 465–495 nm, and it gives off a green light with a wavelength of 515–555 nm. Under UV illumination, cell nuclei that have been labelled with DAPI appear blue. As the kit contains FITC fluorescein, the nuclei of positively apoptotic cells appear green.

### 5.8. Statistical Analysis

The experiments were conducted in triplicate. The results are shown as the mean standard deviation, and deemed statistically significant at *p < 0.05*. The statistical differences were determined using SPSS version 26.0 (SPSS Inc., Chicago, IL, USA).

## Data Availability

Data will be made available on request.

## References

[1] Flynn S., Eisenstein S. (2019). Inflammatory bowel disease presentation and diagnosis. Surg. Clin..

[2] Lukas M. (2010). Inflammatory bowel disease as a risk factor for colorectal cancer. Dig. Dis..

[3] Coward S., Clement F., Benchimol E., Bernstein C., Bitton A., Carroll M., Hazlewood G., Jelinski S., Jones J., Kuenzig E. (2018). Analyzing the rising prevalence of IBD: Predicting the prevalence in 2030 by age group. Gastroenterology.

[4] Ng S.C., Shi H.Y., Hamidi N., Underwood F.E., Tang W., Benchimol E.I., Panaccione R., Ghosh S., Wu J.C., Chan F.K. (2017). Worldwide incidence and prevalence of inflammatory bowel disease in the 21st century: A systematic review of population-based studies. Lancet.

[5] Kaplan G.G. (2015). The global burden of IBD: From 2015 to 2025. Nat. Rev. Gastroenterol. Hepatol..

[6] Benchimol E.I., Bernstein C.N., Bitton A., Murthy S.K., Nguyen G.C., Lee K., Cooke-Lauder J., Siddiq S., Windsor J.W., Carroll M.W. (2019). The impact of inflammatory bowel disease in Canada 2018: A scientific report from the Canadian Gastro-Intestinal Epidemiology Consortium to Crohn’s and Colitis Canada. J. Can. Assoc. Gastroenterol..

[7] Podolsky D.K. (2002). Inflammatory bowel disease. N. Engl. J. Med..

[8] Kaplan G.G., Ng S.C. (2016). Globalisation of inflammatory bowel disease: Perspectives from the evolution of inflammatory bowel disease in the UK and China. Lancet Gastroenterol. Hepatol..

[9] Kaplan G.G., Ng S.C. (2017). Understanding and Preventing the Global Increase of Inflammatory Bowel Disease. Gastroenterology.

[10] Chorus I. (2001). Introduction: Cyanotoxins—Research for environmental safety and human health. Cyanotoxin.

[11] Campos A., Vasconcelos V. (2010). Molecular mechanisms of microcystin toxicity in animal cells. Int. J. Mol. Sci..

[12] Lone Y., Koiri R.K., Bhide M. (2015). An overview of the toxic effect of potential human carcinogen Microcystin-LR on testis. Toxicol. Rep..

[13] Sedan D., Laguens M., Copparoni G., Aranda J.O., Giannuzzi L., Marra C.A., Andrinolo D. (2015). Hepatic and intestine alterations in mice after prolonged exposure to low oral doses of Microcystin-LR. Toxicon.

[14] Su R.C., Blomquist T.M., Kleinhenz A.L., Khalaf F.K., Dube P., Lad A., Breidenbach J.D., Mohammed C.J., Zhang S., Baum C.E. (2019). Exposure to the harmful algal bloom (HAB) toxin microcystin-LR (MC-LR) prolongs and increases severity of dextran sulfate sodium (DSS)-induced colitis. Toxins.

[15] Yang Y., Wang H., Wang X., Chen L., Liu W., Cai D., Deng S., Chu H., Liu Y., Feng X. (2022). Long-term environmental levels of microcystin-LR expo-sure induces colorectal chronic inflammation, fibrosis and barrier disruption via CSF1R/Rap1b signaling pathway. J. Hazard. Mater..

[16] Fischer A., Hoeger S.J., Stemmer K., Feurstein D.J., Knobeloch D., Nussler A., Dietrich D.R. (2010). The role of organic anion transporting polypeptides (OATPs/SLCOs) in the toxicity of different microcystin congeners in vitro: A comparison of primary human hepatocytes and OATP-transfected HEK293 cells. Toxicol. Appl. Pharm..

[17] Valerio E., Vasconcelos V., Campos A. (2016). New Insights on the Mode of Action of Microcystins in Animal Cells—A Review. Mini Rev. Med. Chem..

[18] Bouaicha N., Maatouk I. (2004). Microcystin-LR and nodularin induce intracellular glutathione alteration, reactive oxygen species production and lipid peroxidation in primary cultured rat hepatocytes. Toxicol. Lett..

[19] Mao L., Kitani A., Strober W., Fuss I.J. (2018). The role of NLRP3 and IL-1β in the pathogenesis of inflammatory bowel disease. Front. Immunol..

[20] Xiang L., Li Y.W., Liu B.L., Zhao H.M., Li H., Cai Q.Y., Mo C.H., Wong M.H., Li Q.X. (2019). High ecological and human health risks from microcystins in vegetable fields in southern China. Environ. Int..

[21] Greer B., Meneely J.P., Elliott C.T. (2018). Uptake and accumulation of Microcystin-LR based on exposure through drinking water: An animal model assessing the human health risk. Sci. Rep. UK.

[22] Lun Z., Hai Y., Kun C. (2002). Relationship between microcystin in drinking water and colorectal cancer. Biomed. Environ. Sci..

[23] Dong S., Zhu M., Wang K., Zhao X., Hu L., Jing W., Lu H., Wang S. (2021). Dihydromyricetin improves DSS-induced colitis in mice via modulation of fecal-bacteria-related bile acid metabolism. Pharm. Res..

[24] Su R.C., Warner E.A., Breidenbach J.D., Lad A., Blomquist T.M., Kleinhenz A.L., Modyanov N., Malhotra D., Kennedy D.J., Haller S.T. (2020). CD40 receptor knockout protects against microcystin-LR (MC-LR) prolongation and exacerbation of dextran sulfate sodium (DSS)-induced colitis. Biomedicines.

[25] Bakr A., Alzain M.N., Alzamel N.M., Loutfy N. (2022). Accumulation of Microcystin from *Oscillatoria limnetica* Lemmermann and *Microcystis aeruginosa* (Kützing) in Two Leafy Green Vegetable Crop Plants *Lactuca sativa* L. and *Eruca sativa*. Plants.

[26] Chen J., Xie P., Lin J., He J., Zeng C., Chen J. (2015). Effects of microcystin-LR on gut microflora in different gut regions of mice. J. Toxicol. Sci..

[27] Chen L., Giesy J.P., Xie P. (2018). The dose makes the poison. Sci. Total. Environ..

[28] Jia J., Luo W., Lu Y., Giesy J.P. (2014). Bioaccumulation of microcystins (MCs) in four fish species from Lake Taihu, China: Assessment of risks to humans. Sci. Total. Environ..

[29] Leppkes M., Neurath M.F. (2020). Cytokines in inflammatory bowel diseases–update 2020. Pharmacol. Res..

[30] Tracey D., Klareskog L., Sasso E.H., Salfeld J.G., Tak P.P. (2008). Tumor necrosis factor antagonist mechanisms of action: A comprehensive review. Pharm. Ther..

[31] Blandizzi C., Gionchetti P., Armuzzi A., Caporali R., Chimenti S., Cimaz R., Cimino L., Lapadula G., Lionetti P., Marchesoni A. (2014). The role of tumour necrosis factor in the pathogenesis of immune-mediated diseases. Int. J. Immunopathol. Pharmacol..

[32] Anders H. (2016). Of inflammasomes and alarmins: IL-1β and IL-1α in kidney disease. J. Am. Soc. Nephrol..

[33] Coccia M., Harrison O.J., Schiering C., Asquith M.J., Becher B., Powrie F., Maloy K.J. (2012). IL-1β mediates chronic intestinal inflammation by promoting the accumulation of IL-17A secreting innate lymphoid cells and CD4+ Th17 cells. J. Exp. Med..

[34] Kanneganti T.D. (2017). Inflammatory Bowel Disease and the NLRP3 Inflammasome. N. Engl. J. Med..

[35] Tourkochristou E., Aggeletopoulou I., Konstantakis C., Triantos C. (2019). Role of NLRP3 inflammasome in inflammatory bowel diseases. World J. Gastroenterol..

[36] Zhao D., Fukuyama S., Sakai-Tagawa Y., Takashita E., Shoemaker J.E., Kawaoka Y. (2016). C646, a novel p300/CREB-binding protein-specific inhibitor of histone acetyltransferase, attenuates influenza A virus infection. Antimicrob. Agents Chemother..

[37] Xu X., Li J., Long X., Tao S., Yu X., Ruan X., Zhao K., Tian L. (2021). C646 Protects Against DSS-Induced Colitis Model by Targeting NLRP3 Inflammasome. Front. Pharmacol..

[38] Long X., Yu X., Gong P., Wang X., Tian L. (2022). Identification of WT161 as a Potent Agent for the Treatment of Colitis by Targeting the Nucleotide-Binding Domain-Like Receptor Family Pyrin Domain Containing 3 Inflammasome. Front. Pharmacol..

[39] Martinon F., Mayor A., Tschopp J. (2009). The inflammasomes: Guardians of the body. Annu. Rev. Immunol..

[40] Shi J., Zhao Y., Wang K., Shi X., Wang Y., Huang H., Zhuang Y., Cai T., Wang F., Shao F. (2015). Cleavage of GSDMD by inflammatory caspases determines pyroptotic cell death. Nature.

[41] Alex P., Zachos N.C., Nguyen T., Gonzales L., Chen T.E., Conklin L.S., Centola M., Li X. (2009). Distinct cytokine pat-terns identified from multiplex profiles of murine DSS and TNBS-induced colitis. Inflamm. Bowel Dis..

